# Community-based referral for tuberculosis preventive therapy is effective for treatment completion

**DOI:** 10.1371/journal.pgph.0001269

**Published:** 2022-12-14

**Authors:** Sheela V. Shenoi, Tassos C. Kyriakides, Emily Kainne Dokubo, Vijayanand Guddera, Peter Vranken, Mitesh Desai, Gerald Friedland, Anthony P. Moll

**Affiliations:** 1 Section of Infectious Diseases, Yale School of Medicine, New Haven, Connecticut, United States of America; 2 Yale Institute of Global Health, New Haven, Connecticut, United States of America; 3 Yale School of Public Health, Biostatistics, Yale Center for Analytical Sciences, New Haven, Connecticut, United States of America; 4 U.S. Centers for Disease Control and Prevention, Atlanta, Georgia, United States of America; 5 Philanjalo NGO, Tugela Ferry, South Africa; 6 South African Medical Research Council, Durban, South Africa; 7 U.S. Centers for Disease Control and Prevention, Pretoria, South Africa; 8 Division of Global HIV & TB, U.S. Centers for Disease Control and Prevention, Atlanta, Georgia, United States of America; Zuckerberg San Francisco General Hospital and Trauma Center, UNITED STATES

## Abstract

Expansion of tuberculous preventive therapy (TPT) is essential to curb TB incidence and mortality among people with HIV (PWH), yet implementation has been slow. Innovative strategies to operationalize TPT are urgently needed. Here we present an evaluation of community-based identification and referral of PWH on completion of a six-month course of isoniazid in a highly prevalent region in rural South Africa. Using a community-based TB/HIV intensive case finding strategy, a team of nurses and lay workers identified community members with HIV who were without fever, night sweats, weight loss, or cough and referred them to the government primary care clinics for daily oral isoniazid, the only available TPT regimen. We measured monthly adherence and six-month treatment completion in the community-based identification and referral (CBR) group compared to those already engaged in HIV care. Adherence was measured by self-report and urine isoniazid metabolite testing. A multivariable analysis was performed to identify independent predictors of TPT completion. Among 240 participants, 81.7% were female, median age 35 years (IQR 30–44), and 24.6% had previously been treated for TB. The median CD4 count in the CBR group was 457 (IQR 301–648), significantly higher than the clinic-based comparison group median CD4 of 344 (IQR 186–495, p<0.001). Independent predictors of treatment completion included being a woman (aOR 2.41, 95% 1.02–5.72) and community-based identification and referral for TPT (aOR 2.495, 95% 1.13–5.53). Among the CBR group, treatment completion was 90.0%, an absolute 10.8% higher than the clinic-based comparison group (79.2%, p = 0.02). Adherence was significantly greater in the CBR group than the clinic-based comparison group, as measured by self-report (p = 0.02) and urine isoniazid testing (p = 0.01). Among those not on ART at baseline, 10% of eligible PWH subsequently initiated ART. Community members living with HIV in TB endemic regions identified and referred for TPT demonstrated higher treatment completion and adherence compared to PWH engaged for TPT while receiving clinic-based care. Community-based identification and referral is an innovative adjunctive strategy to facilitate implementation of TB preventive therapy in people living with HIV.

## Introduction

Tuberculosis (TB) is the leading cause of infectious death worldwide and among people living with HIV (PWH) [1]. South Africa has the highest global prevalence of TB/HIV coinfection, particularly KwaZulu-Natal province, with 65–85% of patients with newly diagnosed TB also HIV coinfected [2, 3]. World Health Organization-endorsed TB preventive therapy (TPT) reduces TB incidence by 60–70% and significantly reduces mortality among PWH, independent of antiretroviral therapy (ART) [4–7]. South Africa incorporated TPT into national guidelines in 2010, accounting for the majority of global TPT uptake [8, 9]. Widespread implementation lags in resource-limited settings, requiring innovative strategies to operationalize TPT to reduce morbidity, mortality, and the global TB reservoir [10, 11].

TPT implementation faces multiple patient-level and systemic barriers along the cascade of care; initiation, adherence, and completion rates are suboptimal [11–17]. Community-based strategies for engaging individuals in HIV and TB services have proven successful and valuable [18–29], while modeling and cost effectiveness studies have demonstrated favorable impact on HIV and TB incidence and mortality [30–32]. Transmission in community settings contributes substantially to TB propagation, suggesting that interventions such as active TB case finding and TPT eligibility determined beyond the historic foci of household contacts and health care facilities are important to epidemic control [33–36]. We sought to determine the benefit of an innovative community-based approach for identification and referral (CBR) on TPT completion and adherence, compared to traditional HIV clinical care in resource-limited high TB/HIV burden areas.

## Methods

### Setting

This study was conducted in the rural sub-district of Msinga in KwaZulu-Natal province, an area covering 2000 square kilometers, and a population of 180,000 traditional Zulu people. Msinga is among the most impoverished regions in the country [37, 38]. Residents live in isolated family compounds of traditional Zulu huts, often without ventilation, low levels of electricity (61%) and clean water (69%), and with high levels of unemployment (85%) [37]. Adult HIV prevalence (27%) and drug-susceptible TB (1100/100,000) incidence are extremely high, the latter with 70% HIV coinfection [39]. Transportation is challenging due to the rugged terrain and unpaved roads requiring vehicles with high ground clearance. The provincial district hospital, 16 primary care clinics and three mobile clinics serve the region. TB specimens may be collected at primary care clinics, but all diagnostics, including GeneXpert MTB/RIF^®^ (Cepheid, Sunnyvale, CA, USA) and chest radiography, are only available at the district hospital [18].

### Procedures

From 2013–2016, a team of health educators, nurses, and HIV counselors attended community-based congregate settings, such as bus stations, municipality events, and pension pay points (social grant distribution sites), to provide integrated TB/HIV screening services [18]. The team provided health education on a variety of topics, and offered HIV and TB screening services consisting of rapid fingerstick HIV testing with confirmatory rapid testing if positive following national guidelines [40], WHO-endorsed four symptom screen, and if symptoms reported, sputum collection for GeneXpert testing [18, 41, 42]. Phlebotomy for CD4 cell count was offered, testing was performed on whole blood on by National Health Laboratory Services at the government district hospital, and results were used to determine eligibility for ART according to South African guidelines, which changed during the course of the study [43]. Guidelines expanded access to ART from those with CD4<350 cells/mm^3^ to those with CD4<500 cells/mm^3^ in January 2015 [40]. TPT consisted of oral isoniazid daily for six months. Tuberculin skin testing was recommended to determine TPT duration but had not been implemented [44]. PWH ≥18 years with negative TB symptom screen [40, 42] were informed about TPT including the purpose, dosing, duration, adverse effects, availability at local facilities, and referred to their local primary care clinic.

PWH who were referred and subsequently linked to care, defined as a clinic visit for TPT, at one of five study clinics in Msinga were offered written informed consent. For every enrollee in this CBR group, a PWH ≥18 years initiating TPT in routine HIV care during the same week and clinic, was offered written informed consent to enroll into a clinic-based comparison (CBC) group. After providing consent, all participants received TPT education and medications were subsequently prescribed and dispensed by Department of Health personnel to be taken daily for six months, without routine pyridoxine supplementation or hepatic function monitoring, and integrated with HIV services [44]. Enrollment began July 2013 and all follow up visits and data collection were completed in August 2016. Tuberculin skin testing (TST) was recommended in the South African national guidelines at that time to determine duration of TPT but had not been implemented by the facilities at the time of this study [44]. Study visits were scheduled concurrently with each monthly visit for medication refills. Sociodemographic data, as well as history of prior tuberculosis treatment, cost of transport to the clinic, and alcohol use were elicited. Alcohol consumption was assessed using the AUDIT tool; harmful levels of drinking were defined as a score ≥8 in men and ≥6 in women [45, 46]. Adherence was assessed monthly using two measures—a validated 7-day recall tool (i.e. none, few, half, most, all pills in the last week) [47, 48] and a point of care urine test for isoniazid metabolites (IsoScreen GFC Diagnostics, Oxfordshire, UK), based on the Arkansas colorimetric assay [49–51]. Urine metabolite testing was scored as ‘1’–yellow: no INH, ‘2’–green: INH present, or ‘3’–blue-purple: INH ingestion within the last 24–48 hours. All participants in the CBR and clinic-based comparison group received TPT adherence counseling by the same staff at each visit.

The primary outcome was six month TPT completion [44], defined as pickup of 6 months of medications. TPT completion was compared in the community-based referred (CBR) group and the clinic-initiated comparison group using a non-inferiority design, where the study assesses whether the CBR group is not less successful than the clinic-initiated group. The sample size was calculated based on TPT treatment completion, with a 15% non-inferiority margin, anticipated 85% retention in care, and two-sided alpha of 0.05. With a minimum sample size of 120 participants per group, the study had 95% power to detect non-inferiority.

### Analysis

Descriptive statistics were used to characterize the population and bivariate analyses were performed to identify factors associated with completing TPT. Comparisons between the two groups were made using parametric (t-test) or non-parametric equivalent (for continuous variables) or by chi-square (categorical variables). Variables that were significant at the p<0·2 level in univariate logistic regression models predicting the primary outcome (TPT completion) were entered into a stepwise multivariable logistic regression analysis. Unadjusted and adjusted odds ratios and 95% confidence intervals were calculated. Self-reported adherence was defined as a participant reporting taking their pills. The five categories of self-reported adherence were assigned a score of 0 (’No/Few/Half TPT pills per day’) or 1 (’Most/All TPT pills per day’). Using urine metabolite testing, adherence was assessed as strong if the urine result was blue-purple, indicating isoniazid intake in the last 24-48hours. Strict adherence was defined as blue-purple urine at all of the six follow-up visits. Self-report was also correlated with urine testing scores. For all analyses, a p-value <0.05 was considered statistically significant. All statistical analyses were performed using SAS 9·3 (SAS Institute, Cary NC).

Ethical approval was obtained from the institutional review boards at the South African Medical Association and Yale University School of Medicine. The protocol was also reviewed in accordance with the U.S. Centers for Disease Control and Prevention (CDC) human research protection procedures and was determined to be research, but CDC investigators did not interact with human subjects or have access to identifiable data or specimens for research purposes.

## Results

**Fig 1:**
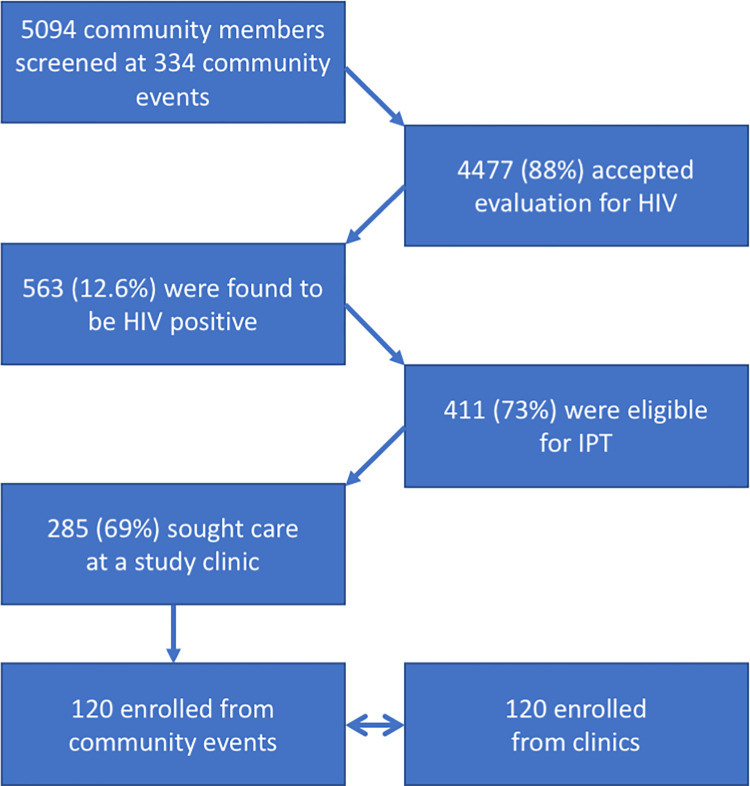
Flow chart depicting community-based TB/HIV screening to enrollment.

During 334 community-based congregate site visits, 563 individuals were identified as HIV positive. Of those, 411 (73%) had a negative TB symptom screen, deemed eligible, and referred for TPT care (Fig 1). Among PWH referred for TPT, 285 (69%) sought care at one of five study clinics; 120 were enrolled and 120 patients already engaged in HIV care and initiating TPT at the same clinic during the same week were enrolled in the comparison group. Participants were predominantly (81.7%) female (Table 1), with a median age of 35 years (Interquartile Range, (IQR) 30–44). Among 209 (87%) with available CD4 values at baseline, the median CD4 count was 417 cells/mm^3^(IQR 238–588). Women had a median CD4 count of 439 (IQR 255–619), while men had a median CD4 count of 393 (IQR 205–514), with no significant difference in CD4 based on gender (p = 0.47). Among the CBR group, the median CD4 count was 457 (IQR 301–648), significantly higher than the clinic-initiated comparison group whose median CD4 cell count at baseline was 344 (IQR 186–495, p<0.001*). Approximately one quarter of participants in the CBR group and comparison group had been previously diagnosed with TB (p = 0.65), and the proportion with harmful drinking was higher in those identified in the community (8.3%) as compared to persons identified at the clinic (3.2%, p = 0.09).

**Table 1:** Characteristics of study participants (n = 240).

	Comparison group (n = 120)	CBR (n = 120)	p-value
Median Age (years)	32 (IQR 27–39)	39 (IQR 31–48)	<0.001*
Female Gender	104 (87%)	92 (76.7%)	0.05^&^
Median Transport Cost to Clinic (one-way, USD)	$0.94	$0.98	0.08*
Elevated AUDIT Score (≥6 for women, ≥8 for men)	4 (3.3%)	10 (8.3%)	0.09^&^
Prior TB	28 (23.3%)	31 (25.8%)	0.65^&^
Median CD4 count cells/mm3 at enrollment	344 (IQR 186–495)	457 (IQR 301–648)	<0.001*
CD4 count ≤200 cells/mm^3^(n = 211)	28(26.2%)	14(13.7%)	0.03^&^
CD4 count ≤350 cells/mm^3^(n = 211)	54 (50.5%)	30 (29.4%)	0.002^&^
CD4 count ≤500 cells/mm^3^ (n = 211)	81 (75.7%)	59 (57.8%)	0.006^&^
On ART at enrollment	85 (70.8%)	60 (50%)	0.001^&^
• Median days of ART	• 442 (IQR 53–1223)	• 1398 (IQR 970–2350)	
On ART by end of study	95 (80.5%)	71 (60.7%)	0.0008^&^

*Wilcoxon two sample test.

^&^chi-square test.

The majority of clinic TPT initiators (70.8%) were already on ART, and had been on treatment for a median of 442 days (IQR53-1223) days. Significantly fewer CBR participants (50%) were taking ART at baseline (p = 0.001), but were on treatment for a median 1398 days (IQR 970–2350).

A comparison of the primary outcome of TPT completion was performed between the two groups (Table 2). Among the CBR group, treatment completion was 90.0%, an absolute 10.8% higher rate than in the clinic-initiated comparison group, where completion was 79.2% (p = 0.03). Overall, 37 (15.4%) participants did not complete TPT, disproportionately higher among clinic initiators (n = 25/120, 20.8%) compared to the CBR group (n = 12/120, 10%), who exhibited significantly (p = 0.03) less loss-to follow up. Among those who did not complete the TPT course (n = 37), 3 (8.1%) were due to side effects and 2 (5.4%) participants died during the course of the study; both were in the CBR arm and their causes of death (stroke, gunshot wounds) were not associated with study participation.

**Table 2:** TPT completion & adherence.

	Comparison Group (n = 120)	CBR (n = 120)	p-value
TPT completion	95 (79.2%)	108 (90%)	0.02^&^
Non-completion	25 (20.8%)	12 (10%)	
Proportion with strict adherence across 6 visits			
Self-report	65 (54.2%)	84 (70%)	0.02^&^
Urine Isoniazid	49 (40.8%)	68 (56.7%)	0.01^&^

^&^chi-square.

Study participants reported taking all or most of their pills during 97.9% (1228 of 1254) patient visits. Of the CBR group, 70% self-reported strict adherence, as compared to 54.2% of the comparison group (p = 0.02). Urine metabolite testing was performed in 97% (1216 of 1254) study visits, and of these, 95.8% (1165 of 1216) were blue-purple, demonstrating strong adherence to TPT. Strict adherence over the TPT course in the CBR group was demonstrated in 56.7% by urine testing, significantly greater than the 40.8% in the comparison group (p = 0.01). Self-reported adherence was positively correlated to urine testing (correlation coefficient of 0.88, p<0.0001).

Among 60 CBR participants not receiving ART at baseline, 28 were eligible or became eligible during the course of the study, and among these, 12 (42.9%) participants initiated ART. Among 35 participants in the comparison group not on ART at enrollment, 14 were eligible or became eligible and 8 (57.1%) initiated ART prior to the end of the study. There was no significant difference in ART initiation between these groups.

Bivariate analyses demonstrated that gender, CD4 count ≤ 350 cells/mm^3^, and community-based referral were associated with TPT completion, whereas concurrent ART, either at baseline (p = 0.90) or initiated over the course of the study (p = 0.34), was not associated with TPT completion. Multivariable stepwise regression analysis identified female gender and community-based referral as independent predictors of TPT completion while CD4 count ≤ 350 cells/mm^3^ dropped out of the model (Table 3).

**Table 3:** Predictors of TPT completion.

	Unadjusted OR (95% CI)	Adjusted OR (95% CI)	p-value
Age	1.04 (0.97–1.04)		
Female Gender	1.84 (0.82–4.15)	2.41(1.02–5.72)	0.042
Transport Cost to Clinic	1.01(0.97–1.05)		
Elevated AUDIT Score	0.99(0.89–1.10)		
Household TB Contact	0.53(0.06–4.99)		
Prior TB	0.86(0.39–1.90)		
Baseline CD4 cells/mm3	1.0(0.99–1.0)		
Baseline CD4 count ≤200 cells/mm^3^	0.65(0.28–1.51)		
Baseline CD4 count ≤350 cells/mm^3^	0.54(0.26–1.13)		
Baseline CD4 count ≤500 cells/mm^3^	0.82(0.37–1.83)		
On ART at baseline	1.05(0.51–2.14)		
On ART at end of study	1.44(0.68–3.05)		
Community based referral for TPT	2.37(1.13–4.97)	2.495(1.13–5.53)	0.036

## Discussion

We compared two strategies for initiating TPT in a high HIV and TB prevalence community in rural South Africa. We demonstrate that a community-based approach to identifying and referring TPT-eligible PWH resulted in a 10% absolute higher completion rate and significantly better adherence compared to those PWH who were already engaged in care. This observed effect was independent of CD4 count on entry into the study. This is among the first studies to successfully leverage a community-based approach for implementing TPT for PWH. The difference in TPT treatment adherence and completion between groups may be due to a number of factors. PWH identified in the community and referred for TPT, often not yet taking ART, may have different perspectives and motivations on their health status and how best to improve their health compared to PWH in the comparison group who were already engaged in HIV care and were largely already taking ART. A higher proportion of PWH engaged in clinic-based care were taking ART and may not prioritize TPT as highly as ART or were impacted by pill burden, resulting in selective adherence to ART over TPT [13, 52]. Thus, PWH in the community may have been particularly motivated to obtain TPT, particularly if not yet eligible for ART. While health care workers may not support or be knowledgeable about the benefit of TPT, this would not differentially affect the study groups [15]. Increased awareness of the effectiveness of TPT in preventing TB incidence and reducing mortality and recommendations for TPT provision to PWH is needed to facilitate widespread implementation. We speculate that community-based engagement results in a potentially different relationship with health workers and subsequent perspective on health care, possibly leading to different motivations to engage in care [53, 54]. Furthermore, individuals identified in the community engaged specifically about TPT may have received more education about TPT than patients in the clinic. However, these are speculative; the study was not designed to answer this question and requires further rigorous evaluation.

Community-based strategies have been successful in identifying individuals with previously unknown or known communicable and non-communicable diseases, and linking them to care [18, 22, 25, 26, 55, 56]. HIV services, particularly in resource-limited settings by NGO partners as well as government programs, have long incorporated community approaches while rigorous evaluations have demonstrated success in improving diagnosis, linkage to care, and initiation of ART [18, 25, 27, 57]. Prevention services to date have primarily emphasized implementing universal HIV test and treat, with a focus on treatment as prevention, as well as medical male circumcision [58–62]. Modeling strategies have demonstrated the benefit of such approaches on HIV incidence and mortality and have demonstrated cost effectiveness [30–32]. Such alternative or differentiated care models need to be employed to improve implementation of preventive strategies, including TPT [63, 64]. Innovative studies are now underway evaluating the benefit of community-based approaches for TPT [62, 65].

Gender had a large impact on outcomes in this study. Women were more successful in completing the TPT course, compared to men, independent of CD4 count. This is not unexpected given that women are more likely to undergo testing, link to care, initiate ART, and overall experience better TB and HIV outcomes than men, particularly in resource limited settings [66–69]. This study contributes to the sparse literature on gender disparities in TB preventive therapy completion [70, 71], suggesting that like HIV retention and TB treatment completion, the TB prevention cascade may differ by gender. Similar to antiretroviral therapy [72], men lag in initiating TB preventive therapy as well. Prevention efforts may be enhanced with gender-specific interventions, such as community case finding targeting men, or adherence clubs targeting men receiving TPT, incorporating gender and cultural norms.

Community-based approaches may also likely support medication adherence. In contrast to other studies, adherence was excellent among all enrollees, potentially attributable to the education and counseling about TPT in both arms. National guidelines [44] do not specify counseling or education, and our overall high rates of completion even among clinic patients may be attributable to increased understanding about the importance of this preventive strategy [13]. The participants in the community-based referral group were significantly more likely to report higher adherence and significantly more likely to have higher isoniazid metabolites in urine. Both groups received the same literacy sessions from the clinic staff and regular counseling at each study visit from study staff. The reasons for better adherence in one group could be related to concurrent ART, however, in our study, concurrent ART use was not an independent predictor of TPT adherence. Though ART use at enrollment was significantly lower in the CBR group, newly prescribed TPT in addition to more recent ART initiation in the comparison group may have contributed to difficulties with TPT adherence.

Among those not receiving ART at enrollment, nearly 10% subsequently initiated ART during the study follow up period. In this instance, engaging community members for preventive therapy also contributed to ART initiation. While the proportion subsequently initiating ART was not statistically significant between both groups, the proportion of those referred from the community for TPT and then subsequently initiating ART was substantial. With the removal of CD4 criteria for ART eligibility and implementation of differentiated care strategies such as community-based ART provision, ART initiation would likely be higher. Another notable finding was the significantly higher CD4 count at enrollment among the CBR group, demonstrating entry into HIV care at an earlier stage of disease accompanied by the potential benefit of longer disease-free survival. This type of community-based ‘hook’ into the health care system can be a valuable tool to engaging individuals into clinical care. In the era of ‘test and treat’, the emphasis remains on rapid ART initiation, but TPT and ART are necessary companions in HIV care, and patients will benefit from bundled care [5, 73]. Laudably, South African guidelines have recently integrated TPT with ART initiation. CBR may facilitate linkage and initiation of either or both TPT and ART, especially if coupled with community-based provision. Though new regimens are becoming available which will facilitate TB preventive therapy adherence and completion, the first step remains initiation. Novel strategies that can effectively engage individuals for integrated HIV and TB care are essential.

There are a number of limitations in this study. Community members referred for TPT were not traced to other than the five study sites, limiting ascertainment of TPT outcomes for referred community members who may have gone to a different facility. Study participants may also represent those particularly motivated to obtain TPT. Secondly, some PWH who were lost to follow-up due to moving out of the area may have continued TPT after relocating; it is possible that if all completed their TPT course, the completion rates would have been similar. Next, urine metabolite testing is limited by detection only over the prior 48–72 hours, and enrollees expecting to be tested may have altered their behavior just prior to an upcoming appointment [49, 50]. Regardless, such a point of care tool is useful to measure adherence, provides rapid patient feedback, and may facilitate adherence monitoring in routine care [74]. Next, the South African government has made progress on implementing TPT, and the clinic environment and knowledge among clinic staff may be different currently than during the study period. Lastly, despite the availability of new shorter regimens, isoniazid remains the current TPT regimen in South Africa and much of the world [1] and the results of this study remains relevant to current global practice. Community-based approaches are valuable regardless of TPT regimen used and offer evidence for improving PWH engagement and strengthening TPT and ART implementation in the current global context.

## Conclusion

TB preventive therapy is an effective strategy to reduce TB incidence in PWH and a WHO-recommended approach to reduce the global burden of TB. Despite being recommended for more than a decade, implementation has not reached optimal levels. Novel strategies such as community-based case finding and referral, designed here to engage individuals for TPT, can be implemented more broadly to engage individuals in care. With updated WHO recommendations on who should receive TPT, including HIV negative adult household contacts of TB patients and other immunosuppressed patients, community-based strategies can offer an innovative complementary approach to traditional TB contact tracing by identifying additional at risk individuals with and without HIV to expand implementation [6, 27, 75]. Community-based identification and referral is an innovative adjunctive strategy for TB prevention to facilitate TB epidemic control.
